# OD14 Harmonization Of HTA For Digital Health Technologies: The EU-Funded European Digital Health Technology Assessment Project

**DOI:** 10.1017/S026646232400148X

**Published:** 2025-01-07

**Authors:** Emmanouil Tsiasiotis, Rossella Di Bidino, Dario Sacchini, Americo Cicchetti

## Abstract

**Introduction:**

The European Digital Health Technology Assessment (EDiHTA), an EU-funded project, aims to deliver the first European digital HTA framework co-created by all relevant stakeholders, validated and ready to use. It responds to the need to harmonize tools, methods, guidelines, and frameworks that are already adopted or under development. All types of digital health technologies (DHTs) and Technology Readiness Levels (TRLs) are covered.

**Methods:**

The EDiHTA project adopts a multistakeholder, multidomain, and modular approach to reach a consensus on a common HTA framework for DHTs (e.g., mApp, telemedicine, artificial intelligence). The currently available HTA frameworks refer to different DHTs (e.g., MAST, DiGA, MAS-AI), include different HTA domains, respond to the needs of different stakeholders, and are applicable in specific local and national contexts. In the majority of cases, they are applicable only in advance TRL. In a few cases, they are already adopted in regulatory/HTA processes. The lack of coordination and harmonization has significant implications for all stakeholders, manufacturers and patients included.

**Results:**

The EDiHTA project adopts a holistic approach involving all relevant stakeholders for consensus building at national and European levels. Cross-border collaboration among stakeholders will be reinforced by the definition of a common taxonomy based on consensus building, and the development and validation of common methods and the HTA framework. Evidence requirements will be defined and made more transparent to increase support for health technology manufacturers. In addition, the harmonization effort, together with the development of a digital platform for the HTA framework, will allow faster and safer access to DHTs by patients/citizens.

**Conclusions:**

The goal of the EDiHTA project is to provide the first European digital HTA framework for DHTs that will be adopted by all relevant stakeholders along the lifecycle of digital technologies to promote sustainable health systems. A consortium comprising 15 partners from eight countries, including all relevant stakeholders, works to reach these goals within the next four years.

